# Microarray analysis of choroid/RPE gene expression in marmoset eyes undergoing changes in ocular growth and refraction

**Published:** 2008-08-11

**Authors:** Lilian Shelton, David Troilo, Megan R Lerner, Yuriy Gusev, Daniel J Brackett, Jody Summers Rada

**Affiliations:** 1 Departments of Cell Biology, University of Oklahoma Health Science Center, Oklahoma City, OK; 2 Department of Biomedical Science and Disease, The New England College of Optometry, Boston, MA; 3 Department of Surgery, University of Oklahoma Health Science Center, Oklahoma City, OK

## Abstract

Purpose: Visually guided ocular growth is facilitated by scleral extracellular matrix remodeling at the posterior pole of the eye. Coincident with scleral remodeling, significant changes in choroidal morphology, blood flow, and protein synthesis have been shown to occur in eyes undergoing ocular growth changes. The current study is designed to identify gene expression changes that may occur in the choroid/retinal pigment epithelium (RPE) of marmoset eyes during their compensation for hyperopic defocus as compared to eyes compensating for myopic defocus.

Methods: Total RNA was isolated from choroid/RPE from four common marmosets (*Callithrix jacchus*) undergoing binocular lens treatment using extended wear soft contact lenses of equal magnitude but opposite sign (±5 diopter [D]). After reverse transcription, cDNA was labeled and hybridized to a human oligonucleotide microarray and gene transcript expression profiles were determined. Real-time polymerase chain reaction (PCR) and western blot analysis were used to confirm genes and proteins of interest, respectively.

Results: Microarray analyses in choroid/RPE indicated 204 genes were significantly changed in minus lens-treated as compared with plus lens-treated eyes (p<0.05, Student’s t-test). Differential choroid/RPE expression of protein tyrosine phosphatase, receptor type, B (*PTPRB*), transforming growth factor beta-induced (*TGFBI*), and basic fibroblast growth factor 2 (*FGF-2*) were confirmed by real-time PCR. TGFBIp was confirmed at the protein level by western blot analysis in marmoset and human cornea, choroid/RPE, and sclera.

Conclusions: The present study demonstrated that significant gene expression changes occur in the marmoset choroid/RPE during visually guided ocular growth. The identification of novel candidate genes in choroid/RPE of marmoset eyes actively accelerating or decelerating their rates of ocular elongation may elucidate the choroidal response during the regulation of postnatal ocular growth and may lead to the identification of choroid/RPE signaling molecules that participate in scleral remodeling.

## Introduction

Myopia is a common abnormal visual condition in which the eye is too long for its optical power, resulting in the focal plane being located in front of the retina. Generally, vitreous chamber elongation stabilizes after puberty; however it fails to stabilize in some cases. As a result of increased ocular elongation, the retina thins and stretches to cover the eye which can result in retinal detachment leading to blindness in extreme cases [1]. Animal models of myopia have provided significant insights into the cellular and molecular events underlying the control of ocular elongation and the refractive state. Depriving the retina of form vision by lid suture, translucent occluders, or by imposing hyperopic defocus with the use of negative power concave (minus) lenses [2,3] results in an accelerated rate of axial elongation and the development of myopia. In contrast, exposure to myopic defocus, as a result of restoration of unrestricted vision in previously form-deprived eyes (recovery), or treatment with positive power convex (plus) lenses results in a decelerated rate of axial elongation and hyperopia as compared with the contralateral untreated eye. Significant changes in scleral extracellular matrix synthesis, accumulation and turnover are associated with changes in vitreous chamber elongation rates during the development of experimentally induced myopia or hyperopia in a variety of animals [4]. Changes in scleral proteoglycan synthesis [4,5], matrix metalloproteinase-2 activity [6-8], collagen synthesis, the rate of collagen degradation [5,9], and changes in expression in transforming growth factor-β (TGF-β) 1, 2, and 3 [8,10,11] have all been identified to occur in scleras during visually induced changes in ocular growth. Although the mechanisms underlying visually guided changes in ocular elongation have yet to be determined, it is widely accepted that local factors play an important role in these sclera remodeling events [12].

The choroid is the highly vascularized layer located between the retina and the sclera, and has been implicated in the regulation of scleral metabolism. Studies have shown that the chick choroid undergoes a rapid and dramatic increase in thickness during the recovery from induced myopia [13,14]. Similar, but much smaller choroidal thickness changes have been demonstrated in marmoset and macaque eyes recovering from experimental myopia [15,16]. These thickness changes may be the result of changes in choroidal blood flow [17] and vascular permeability [18-20]; changes in the production of osmotically active molecules, such as glycosaminoglycans, that draw water into lymphatic lacunae present in the choroidal stroma [13,21]; or by the contraction and relaxation of nonvascular smooth muscle cells present within the choroidal stroma [22,23]. It is hypothesized that choroidal thickening is a rapid mechanism for reducing myopia, by pushing the retina to the focal point [13]. Concomitant with choroidal thickening, the rate of vitreous chamber elongation slows dramatically as scleral proteoglycan synthesis changes [24]. Moreover, suprachoroidal fluid removed from eyes undergoing recovery following experimentally induced myopic defocus inhibits scleral proteoglycan synthesis in vitro as compared with that of fluid isolated from control choroids [20].

It has also been recently shown that significant differences in RPE gene expression are associated with imposed +10 D and −10 D defocus in chicks and that some of these gene expression changes might modulate the growth of the choroid and sclera in young chick eyes [25]. Based on these observations, we hypothesize that changes in choroid/RPE gene expression occur during visually guided ocular growth. These gene expression changes may be responsible for mediating the choroidal response to optical defocus, as well as for the regulation of scleral matrix remodeling and the rate of vitreous chamber elongation.

In an effort to characterize the molecular mechanisms involved in the response to visually induced changes in ocular growth, we induced interocular growth rate differences and anisometropia in young marmosets (*Callithrix jacchus*) using binocular lenses of the same power but opposite sign (±5 D). Microarray analyses were then used to identify differences in gene expression in choroid/RPE from minus lens-treated eyes as compared with that from plus lens-treated eyes as a first step to uncovering novel choroid/RPE proteins involved in mediating scleral extracellular matrix remodeling and the regulation of ocular growth. In this study, we report gene expression changes and identify the presence of a novel protein in the choroid/RPE of marmoset eyes undergoing active ocular elongation.

## Methods

### Animals and tissue preparation

Marmosets were raised in the animal care facility at the New England College of Optometry (Boston, MA) according to United States Department of Agriculture standard for animal care and use and the ARVO Statement for the Use of Animals in Ophthalmic and Vision Research. Artificial lighting was provided using daylight-balanced fluorescent lamps (Durotest Vita-Light, Philadelphia, PA) on a 12h:12h light-dark cycle. Interocular growth differences were maximized by raising juvenile marmosets (n=4) with extended wear soft contact lenses of equal magnitude but opposite sign (±5 D). All four marmosets (A, B, C, and D) wore lenses for 92 days beginning at either 60 days old (A and B) or 62 days old (C and D). Lenses were removed daily at the beginning of the dark period, and replaced the next morning, just before the light period. Experimentally induced changes in refractive state were measured by retinoscopy and Hartinger refractometry under cycloplegia with 1% cyclopentolate, and reported as the average of the spherical equivalents from both measures. Axial length changes were measured with A-scan ultrasonography and reported as changes in vitreous chamber depth [26]. Ocular growth and refractive measurements were taken every two weeks, and animals were euthanized via an intravenous injection of a lethal dose of pentobarbital (190 mg pentobarbital/100 g) following 92 days of lens wear when it was apparent that vitreous chamber depth and ocular refractions were substantially different in the minus lens-treated eye as compared to the contralateral plus lens-treated eyes.. The choroid/RPE were dissected from the sclera and retina, and placed in Modified Eagle's Medium (MEM) culture medium until snap frozen (5–10 min following dissection). Retinas and choroid/RPE were immediately snap frozen in liquid nitrogen and shipped on dry ice to Jody Summers Rada at the University of Oklahoma Health Sciences Center (Oklahoma City, OK). Tissue was stored at −80 °C until the time of RNA isolation.

### RNA isolation

Total RNA was isolated from the entire marmoset choroid/RPE using TRIZOL Reagent following the standard protocol (Invitrogen, Carlsbad, CA) and RNeasy MinElute CleanUp (Qiagen, Valencia, CA). Briefly, frozen tissues were homogenized in 1 ml TRIZOL Reagent using a VirTis homogenizer (Gardiner, NY) before a 5 min incubation at room temperature and chloroform treatment (200 μl) for dissociation of nucleoprotein complexes. The tubes were vigorously shaken by hand for 15 min before they were incubated at room temperature for 2 min. The upper aqueous (RNA) phase was collected following centrifugation (12,000x g for 15 min at 4 °C). The aqueous phase was then collected into a fresh tube then precipitated into a gel-like pellet with 500 μl isopropyl alcohol by incubating for 10 min at room temperature and centrifuging 12,000x g for 15 min at 4 °C. After removal of the supernatant, the RNA pellet was washed once with 1 ml 75% ETOH by centrifugation at 7500x g for 5 min at 4 °C. The RNA pellet was dried by air for 10 min before redissolving the RNA in 100 μl RNase-free water and incubating for 10 min at 60 °C. RNA was stored at −80 °C until use. RNA concentration and purity was determined at an optical density ratio of 260/280 using the Nanodrop® ND-1000 spectrophotometer (NanoDrop Technologies, Wilmington, DE) and then transferred for microarray analysis to the Facility for Molecular and Cellular Studies of Human Disease at the University of Oklahoma Health Science Center.

To ascertain the effect of dissection, snap freezing, and shipping on the quality of marmoset ocular RNA, total RNA was extracted from the left and right retinas of subject A and was assessed on a RNA 6000 Nano LabChip® kit using an Agilent 2100 bioanalyzer (Agilent Technologies, Inc., Santa Clara, CA; Laboratory for Genomics and Bioinformatics Sequencing Core Facility, University of Oklahoma Health Science Center) by microcapillary electrophoretic RNA separation.

### Microarray

To date, no microarray platform is available using marmoset DNA sequences. Therefore, we used a human cDNA plastic array (Atlas™ Plastic Human 12K Array; Clonetech Labs, Mountain View, CA), which used long (80 bp) oligonucleotide sequences previously characterized in terms of function or disease association. This gene array contains 12,000 genes printed in duplicate on a plastic support surface. Since the DNA sequence homology is relatively high between that of humans and marmosets (85–97% homologous) for a set of annotated genes [27], the use of a long oligonucleotide array allowed for the detection of changes in expression of marmoset genes that are highly homologous, yet not identical to that of humans.

### cDNA probe synthesis from total RNA

Choroid/RPE RNA was digested with DNase I using NucleoSpin RNA spin columns (Clonetech Labs). α-^33^P radiolabeled cDNA probes were generated from total RNA using random primer nucleotides together with PowerScript enzyme (Clonetech Labs), according to the recommended protocol for the BD Atlas Pure Total RNA Labeling System (BD Biosciences Clontech, Heidelberg, Germany). Total RNA (1.55–3.27 μg) for each pair of choroid/RPE's were used for right and left eyes of each subject. Briefly, a master mix (4 μl 5X BD PowerScript reaction buffer, 2 μl 10X dNTP mix, 7 μl [α-33P]dATP

(>2,500 Ci/mmol, 10 μCi/μl), 1.5 μl 100mM DTT) was prepared per reaction at room temperature. For each pair of choroid/RPE, 1.55–3.27 μg of total RNA were combined and gently mixed with 2 μl 100 μM N-15 (random primer mix) and 1 μl cDNA synthesis control (1:200) for single-stranded cDNA synthesis. The same amounts of starting RNA were used for right and left eyes of each subject. Each reaction tube was then incubated at 65 °C for 3 min, and then 42 °C for 2 min. After the addition of 2 μl/reaction PowerScript Reverse Transcriptase to the master mix, 18.5 μl master mix/reaction tube was added, then incubated at 42 °C for 30 min before stopping the reaction by the addition of 3 μl 10X termination mix. cDNA was purified from unincorporated nucleotides by column chromatography (NucleoSpin Extraction Spin column; (Clonetech Labs) and the radioactivity of the probes was determined by scintillation counting.

### Hybridizing cDNA probes to the Atlas array

Separate Atlas arrays were used for cDNA probes generated from choroid/RPE RNA from each of the eight marmoset choroid/RPE RNA samples and placed in individual Atlas hybridization boxes with equal amounts of ^33^P-labeled probe for the right and left eyes of each marmoset. Atlas hybridization boxes with equal amounts of ^33^P-labeled probe for the right and left eyes of each marmoset. Specifically, for marmoset A, 16x10^6^ cpm of radiolabeled cDNA were used from the choroid/RPE probes from right and left eyes; for marmoset B, 13x10^6^ cpm of radiolabeled cDNA were used; for marmoset C, 13x10^6^ cpm of radiolabeled cDNA were used; and for marmoset D, 14x10^6^ cpm of radiolabeled cDNA were used. Plastic arrays were processed for probe hybridization and washed at 68 °C according to the manufacturer’s protocols. After careful removal of the membrane from the rinse, the membranes were allowed to dry at room temperature and placed on phosphorimaging screens (Molecular Dynamics, Piscataway, NJ) for a 72 h exposure. The exposed phosphorimaging screens were digitized using a Storm Phosphorimaging System (Molecular Dynamics).

### Array analysis

The expression levels of individual genes was analyzed and compared (plus lens-treated eyes versus minus lens-treated eyes) using Array Vision Software (Imaging Research Inc., Ontario, Canada). The total amount of hybridization signal emitted from each array locus (discrete hybridization site) was reflected in the density of each array locus on the membrane and expressed as “molecular dynamic counts.” The background or nonspecific hybridization signal was measured from negative DNA control loci on each plastic array and subtracted from the signal of each individual locus. The human 12,000 gene arrays consist of two spots on each plastic array, and the mean of both spots following background subtraction was calculated as the magnitude for the expression of each gene. Gene expression changes were compared between choroid/RPE's of minus lens-treated eyes to that of plus lens-treated eyes. The magnitude of each corresponding gene was calculated and the relative difference in signal of the minus lens-treated eyes to that of the plus lens-treated eyes was expressed either as a ratio of the average values: average Magnitude [minus lens]/average Magnitude [plus lens] or as the difference of the average values: average magnitude [minus lens] – average Magnitude [plus lens]. Genes with significant expression differences between the minus lens-treated eyes and plus lens-treated eyes for the four marmosets were analyzed further for volcano plot and global canonical pathway analyses. Significant differences were determined by Student's t-test using a p-value of ≤0.05.

### Real-time polymerase chain reaction

The same RNA isolated from choroid/RPE of binocular (aniso) lens-treated marmosets was used for real-time PCR analysis. cDNA was generated from total RNA by reverse transcription and real-time PCR was performed as previously described [28]. Briefly, samples were run in triplicate using gene-specific human primers together with SYBR Green (Molecular Probes, Eugene, OR) in a 96 well plate format, using an i-Cycler iQTM Multi-Color Real Time PCR Detection System (Bio-Rad, Hercules, CA). Primers were selected from human sequences of characterized genes using BLAST, Primer3, and Sigma-Genosys (St. Louis, MO), respectively. Primers used in this study included TGF-β inducible gene-h3 (*TGFBI*; also known as *ßigh3*, MP78/70, RGD-CAP, and keratoepithelin), protein tyrosine phosphatase receptor-type B (*PTPRB*), and basic fibroblast growth factor 2 (*FGF-2*). The cyclophilin A gene, peptidylprolyl isomerase A, (*PPIA*), was used as a control to normalize for variation in starting cDNA between samples (Table 1). Human primers for hypoxanthine phosphoribosyltransferase (*HPRT*) and glyceraldehyde 3 phosphate dehydrogenase (*GAPDH*) were also tested on marmoset cDNA samples from choroid/RPE, with inconsistent results. On the other hand, the cyclophilin A primers used in this study consistently generated high quality PCR product with a high efficiency (Table 1). No consistent differences in steady-state mRNA levels for cyclophilin A were noted in our real time PCR analyses of cDNA from marmoset choroid/RPE. Furthermore, while cyclophilin A was not present on the Clontech microarray, cyclophilins B, C, D, E, F, G, and H were present on the microarrays and were not significantly different in choroid/RPE of minus lens-treated eyes as compared with that of plus lens-treated eyes. Primer efficiencies were determined by the method of Pfaffl [29] using marmoset choroid/RPE mRNA.

**Table 1:** Real –Time PCR gene primers, efficiencies and homology of the marmoset PCR product with the human sequence

**Gene**	**GenBank accession number**	**Primer sequence (5’-3’)**	**Product Size** **(bp)**	**Tm (ºC)**	**Primer** **efficiency**	**%** **homology**
*Cyclophilin A*	NM_021130	F: TTT TCA TCT GCA CTG CCA AG	300	57.4	0.93	0.95
		R: GGA AAA CAT GGA ACC CAA AG				
*TGFBI*	M77349	F: GTC CAC AGC CAT TGA CCT TT	373	61	0.92	0.95
		R: GTC TCC CTT CAG GAC ATC CA				
*FGF-2*	NM_002006	F: GGT GAA ACC CCG TCT CTA CA	172	55	0.93	0.78
		R: TCT GTT GCC TAG GCT GGA CT				
*PTPRB*	NM_002837	F: ACA TCC CTG GCA ACA ACT TC	323	60	0.94	0.97
		R: TGG CCA CAC CGT ATA GTG AA				

PCR products were confirmed by DNA sequencing following purification on quick spin columns (Qiaquick; Qiagen) using an ABI 3730 capillary DNA sequencer (Applied Biosystems, Foster City, CA, in collaboration with the Laboratory for Genomics and Bioinformatics Sequencing Core Facility, University of Oklahoma Health Science Center).

Due to its relatively small size, sequencing of the *FGF* PCR product required prior cloning into TOPO^®^ vector using pCR^®^2.1-TOPO-TA cloning kit using standard protocols (Invitrogen). Briefly, 2 µl TOPO® Cloning reaction was added to One Shot^®^TOP10 competent *E*. *coli* and transformed by incubating the mixture on ice, followed by heat-shock at 42 °C. Transformed colonies were identified on ampicillin-selective plates treated with 5-bromo-4-chloro-3-indolyl-b-galactopyranoside (Xgal) and isopropylthio-*B*-D-galactoside (IPTG) following overnight incubatation at 37 °C. Plasmid DNA was purified from transformed bacteria using the Qiagen Plasmid Mini Kit (Qiagen tip-20; Qiagen) using the recommended manufacturer’s protocol. Cloned PCR products were sequenced using M13 primers provided by The Laboratory for Genomics and Bioinformatics Sequencing Core Facility (University of Oklahoma Health Science Center, Oklahoma City, OK).

Relative levels of gene expression were determined between minus lens-treated and plus lens-treated eyes using the mean normalized expression (MNE) values [30]. Correct product size and lack of primer dimer formation was confirmed by DNA agarose-gel electrophoresis and melt curve analysis, respectively.

### Western blot analysis

Total protein was extracted from the cornea, choroid/RPE, sclera, and retina of both marmoset and human eyes by vigorously mixing in 2% SDS, and 10 μl of protein extracts were directly applied to 10% Bis-Tris Gel NuPAGE™ SDS–PAGE gels (Invitrogen). Gel samples were electrophoresed under reducing conditions and electroblotted onto a nitrocellulose membrane using an electro-transfer unit (XCELL Sureback™ Electrophoresis Cell; Invitrogen) or stained with SimplyBlue™ SafeStain (Invitrogen) according to the manufacturer’s instructions. Blots were probed with 100 μg (1 mg/ml) anti-hTGFBIp affinity purified goat IgG antibody (R&D systems; Minneapolis, MN) at a 1:500 dilution in blocking buffer (PBS containing 0.1% Tween-20 and 0.2% I-Block [Tropix, Bedford, MA]) for 4 h at room temperature. After washing, blots were incubated with rabbit anti-goat IgG (whole-molecule) conjugated to alkaline phosphatase (Sigma, St. Louis, MO) at a dilution of 1:1000 for 1 h at room temperature. In some cases, 1 μM anti-hTGFBIp was pre-incubated with human recombinant TGFBIp in blocking buffer prior to use in western blot analyses. Between incubations the blot was washed three times for 10 min with PBS containing 0.05% Tween20. CDP-*Star* ® Ready-to-Use with Nitro-BlockII™ (Tropix) was added to the blot for 5 min and then exposed on film.

### Cell attachment assay

Primary human scleral fibroblasts (HSFs) were prepared and maintained as previously reported [28]. Briefly, HSFs were cultured on 100 mm plates containing Dulbecco’s modified Eagle’s medium (DMEM) containing 1X antibiotic/antimycotic (1X a/a; penicillin-streptomycin/amphotericin B solution; Invitrogen) and 15% fetal bovine serum (FBS) at 37 °C with 5% CO_2_. After reaching confluency, the cells were detached by gentle pipeting following incubation in 0.53 mM EDTA in Hank’s balanced salt solution (HBSS; Sigma-Aldrich) and counted using a hemocytometer.

For cell attachment assays, 50 μl protein (0-25 μg/ml; BSA, fibronectin, or TGFBIp) in phosphate buffered saline (PBS) was added to poly-D-lysine 96 well ProBind™ (Becton Dickinson Labware, Franklin Lakes, NJ) plates in triplicate and allowed to bind for 1 h at 37 °C/5% CO_2_. Next, 100 μl HSFs (2x10^3^ cells/well) were added to each well in serum-limited medium (DMEM with 1X antibiotic/antimyotic containing 0.5% FBS) for 45 min at 37 °C/5% CO_2_. The unattached cells were gently removed by washing twice with PBS, and then 100 μl hexosaminidase substrate (50 mM citrate buffer, pH 5.0 with 3.75 mM N-acetyl-β-D-glucosaminidase and 25% Triton-X-100) was added to each well for 1 h at 37 °C/5% CO_2_. Finally, 150 μl (50 mM) glycine buffer, pH 10.4, with 5 mM EDTA was added to each well to stop enzyme activity, and the absorbance read at 405 nm.

### Statistical analysis

Comparisons of ocular size and refraction between plus lens-treated and minus lens-treated eyes were made using the Student’s *t*-test for matched pairs with the assistance of GraphPad Prism version 4.03 for Windows (GraphPad Software, San Diego, CA). Patterns of gene expression and differentially expressed genes identified through microarray experiments were analyzed using MultiExperiment Viewer version 4.0, developed and maintained by the team at Dana-Farber Cancer Institute (Harvard University Medical School, Boston, MA). Statistical comparisons of gene expression between minus lens-treated and plus lens-treated eyes were conducted by use of paired and unpaired Student’s *t*-tests using Array Vision Software (Imaging Research Inc., Ontario, Canada).

Ingenuity Pathways Analysis (Ingenuity Systems, Redwood City, CA) was used to identify canonical pathways potentially involved in the choroidal and RPE response to both minus and plus lens treatments. The Ingenuity Pathways Analysis knowledge database (IPKB; Ingenuity Systems, Redwood City, CA) is a database developed by review of peer reviewed journals and includes information for proteins, genes, protein-complexes, cells, cellular components, tissues, organs, and disease interrelationships. The database currently covers over 23,900 mammalian genes for human, mouse, and rat and is updated quarterly. Genes that were significantly upregulated or significantly downregulated (p≤0.05) in minus lens-treated eyes as compared with plus lens-treated eyes were subjected to canonical pathways analysis using the IPKB. The significance value assigned to each function was calculated using the right-tailed Fischer’s Exact Test. This test determines the chance that the genes of interest (the significantly upregulated and significantly downregulated genes in choroid/RPE of minus lens-treated eyes as compared with plus lens-treated eyes) participate in known biologic pathways from the Ingenuity Pathways Analysis library of canonical pathways. Fischer’s exact test was used to calculate a p-value, determining the probability that the association between the genes in the data set and the canonical pathway was explained by chance alone. Statistical significance of the effect of *TGFBI* expression on scleral fibroblast attachment was determined using the Student’s *t-*test.

## Results

### Binocular lens treatment

**Figure 1:**
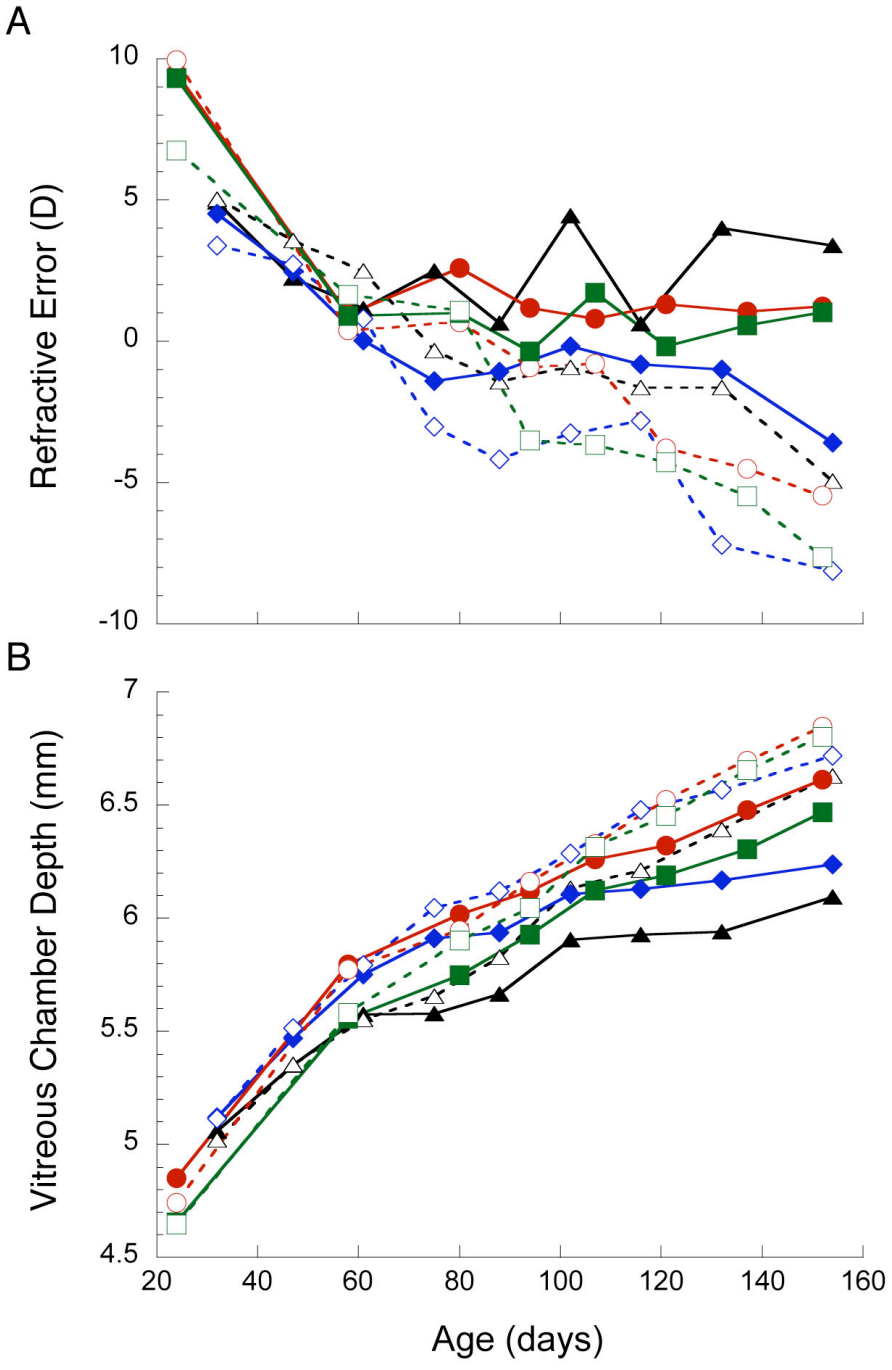
Refractive state and vitreous chamber depth of marmoset eyes following binocular +5 D and -5 D lens treatment over 92 days. **A:** −5 D lens-treated eyes (open symbols) exhibited a more negative refractive error relative to contralateral +5 D lens-treated eyes (closed symbols). **B:** −5 D lens-treated eyes (open symbols) exhibited longer vitreous chambers relative to contralateral +5 D lens-treated eyes (closed symbols). Eyes of the four individual marmosets are represented by different symbols (red circle, subject A; green square, subject B; black triangle, subject C; blue diamond, subject D).

**Figure 2:**
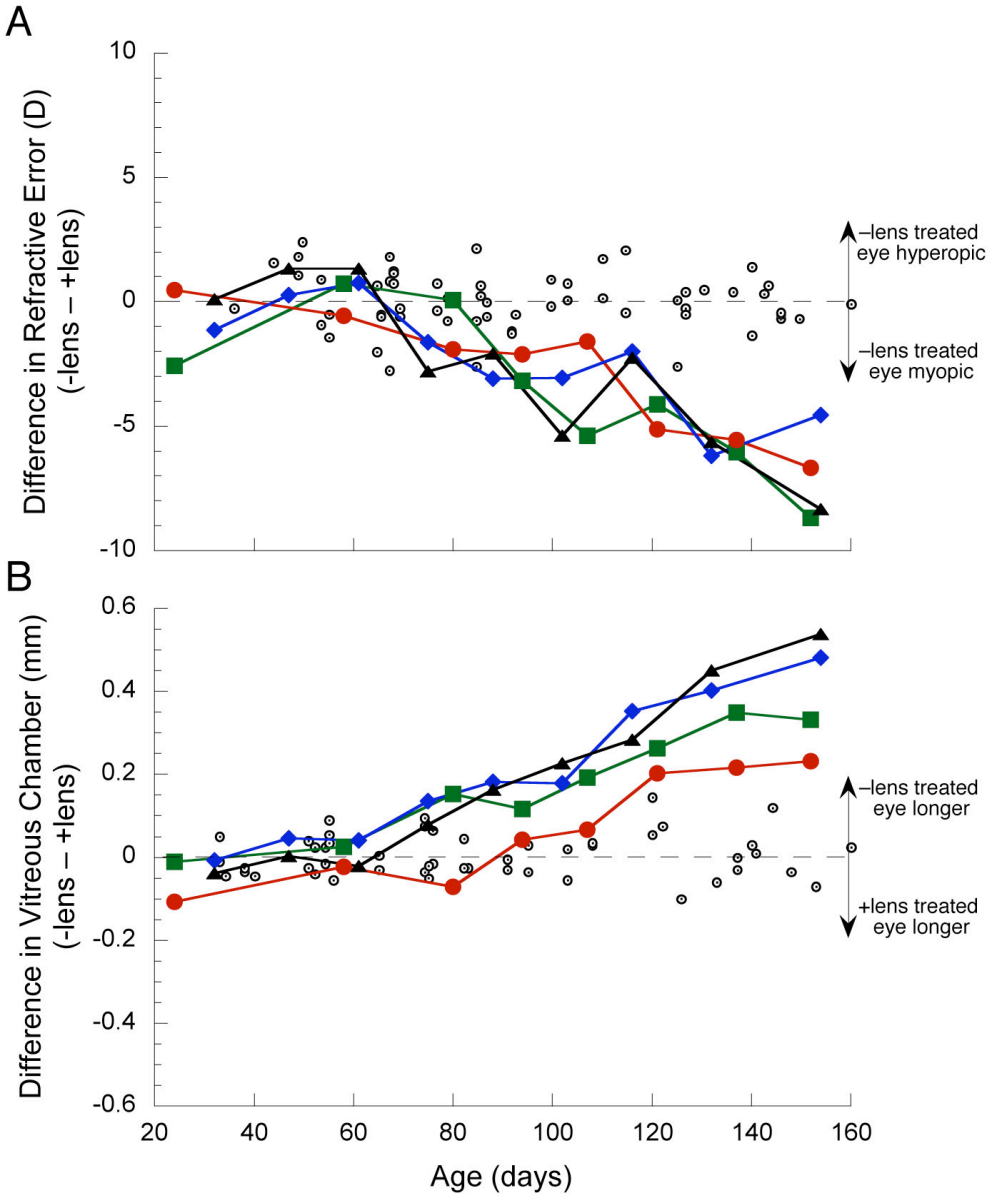
Interocular differences in ocular refraction and vitreous chamber depth in −5 D lens-treated eyes and in contralateral +5 D lens-treated eyes in four individual marmosets. For comparison, interocular differences in refraction (**A**) and vitreous chamber depth (**B**) in two eyes of untreated normal animals (n=30) over the same age range are indicated as dotted circles. Eyes of the four individual marmosets are represented by different symbols (red circle, subject A; green square, subject B; black triangle, subject C; blue diamond, subject D).

Binocular -5/+5 D lens treatment resulted in distinct interocular differences in refractive development (Figure 1A) and vitreous chamber growth (Figure 1B). Following 92 days of binocular lens treatment, minus lens-treated eyes had significantly more myopia (−6.545±0.787 D; Figure 2A) and significantly longer vitreous chamber depth (6.750±0.0478 mm; Figure 2B) as compared with contralateral plus lens-treated eyes (0.505±1.467 D and 6.354±0.116 mm; p<0.01 and p<0.05, respectively; Student’s t-test). For comparison, interocular differences in refraction (Figure 2A) and vitreous chamber depth (Figure 2B) of age-matched untreated animals were included and indicated as separate points. Examination of the effective refractive state (refraction at different points during the rearing period with the lenses in place) indicated that in all animals, the positive-lens-treated eye was always experiencing relative myopia compared to the negative-lens-treated eye (data not shown). No significant changes were detected in anterior chamber depth, lens thickness, corneal curvature, or choroid thickness between minus lens-treated and plus lens-treated eyes at the time of sacrifice.

### RNA isolation

**Figure 3:**
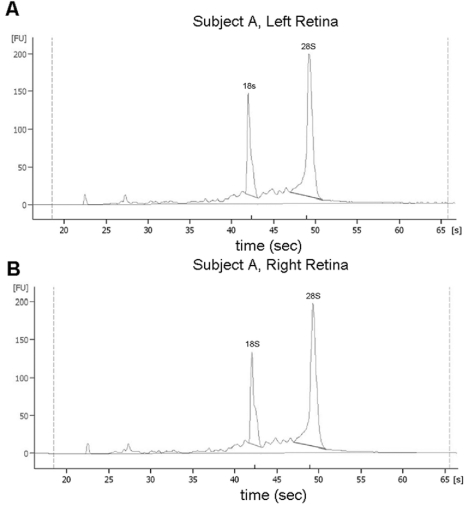
Electropherograms of total RNA extracted from the retinas of left and right marmoset eyes of subject A following microcapillary electrophoresis. Electropherograms demonstrate well defined 18S and 28S rRNA peaks indicating high quality RNA was extracted from the retinas of left (**A**) and right (**B**) marmoset eyes. Since the retinas were dissected and processed in parallel with the choroid/RPE tissue used in the present study, these results suggest that the tissue dissection, snap freezing, and shipping of tissues was adequate to preserve high quality RNA. FU is fluorescence units.

Using standard protocols, Trizol extraction of choroid/RPE tissue from individual marmoset sets yielded approximately 8-10 μg total RNA/eye of high purity (260/280 nm ratios of 1.96-2.08). Measurements of incorporated ^33^P indicated that cDNA probe synthesis from total RNA of each marmoset choroid/RPE was well within the optimal range (5-25x10^6^ cpm/5 μl) following purification by column chromatography for microarray analyses (13-17x10^6^ cpm/5 μl). Moreover, electrophoretic analyses were carried out on total RNA isolated from the left and right retinas of one pair of marmoset eyes (subject A) and shipped and stored in parallel with the choroid/RPE samples. Intact 28S and 18S rRNA subunits were apparent on electropherograms, suggesting that tissue isolation, storage and shipping were suitable to maintain the integrity of mRNA isolated from marmoset eyes used in this study (Figure 3).

### Microarray analysis of marmoset choroid/RPE RNA

**Figure 4:**
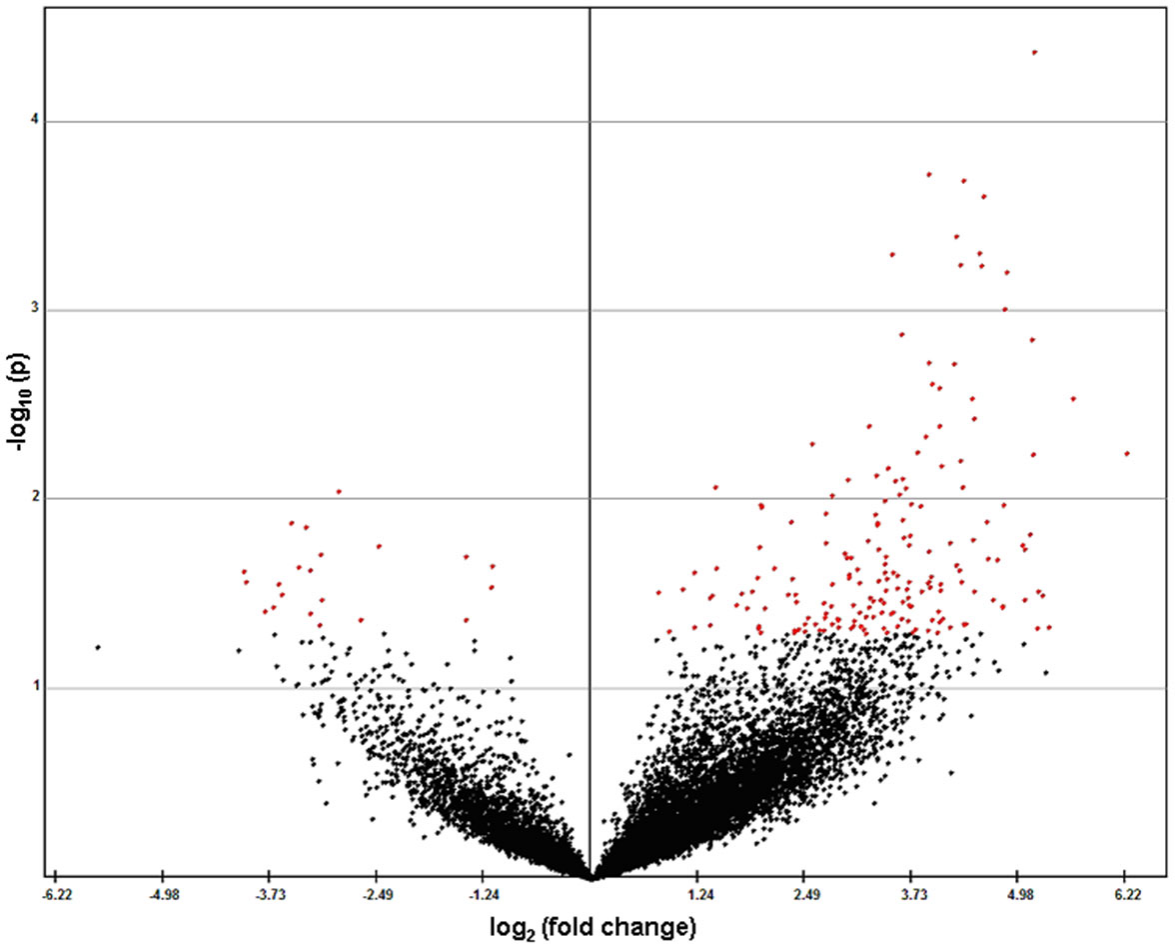
Volcano plot of marmoset choroid/RPE microarray data. Scatter plot represents a summary of the *t*-tests for the individual genes. For each gene this plot shows log_2_ of fold change between the mean expression in minus lens-treated group and plus lens-treated group as plotted against −log_10_ (p), where p is a probability value (for a given gene) which is associated with the Student *t*-test comparison of the two groups of samples. Using a p value of 0.05 as the cutoff threshold, 21 genes were significantly downregulated in minus lens-treated eyes as compared with plus lens-treated eyes (negative log_2_) and 183 genes were significantly uppregulated in minus lens-treated eyes as compared with plus lens-treated eyes (positive log_2_). Each point represents an individual gene in the array; genes not significantly altered are depicted in black, whereas genes significantly changed are indicated in red. Those genes appearing on the upper left or right region have a large fold-change and a smaller p value.

In an effort to identify genes involved in the regulation of ocular growth, microarray analysis was performed to compare choroid/RPE gene expression in marmoset (*Callithrix jacchus*) eyes during treatment with +5 D lenses with that of eyes during treatment with −5 D lenses. A total of 11,867 genes were evaluated in eight separate microarrays of plus lens-treated and minus lens-treated eyes of four marmosets using MultiExperiment Viewer v. 4.0 [31]. Following background subtraction, a total of 8,270–11,360 genes were available for analysis on each of the 8 microarrays. Comparison of differentially expressed genes between the choroid/RPE of right and left eyes indicated that a total of 204 genes were significantly upregulated or downregulated in minus lens-treated eyes as compared with plus lens-treated eyes (p<0.05; Student’s *t*-test for unmatched pairs). Of these, 183 genes were significantly upregulated and 21 genes were downregulated in minus lens-treated eyes as compared with plus lens-treated eyes (Figure 4**)**. Choroid/RPE gene expression differences between minus lens-treated and plus lens-treated eyes, together with the Student’s *t*-test criteria (p<0.05) were summarized by volcano plot analysis (Figure 4). The volcano plot served as a visualization tool for presentation of the *t*-test results when the two groups of samples were compared. For each gene, this plot demonstrated the log_2_ of the fold change in the average expression of the two groups (+5 D lens-treated eyes and −5 D lens-treated eyes) as plotted against −log10(p). P represents the probability value for a given gene which is associated with the t-test comparison of the two groups of samples. Genes with statistically significant differential expression according to the gene-specific *t-*test were found above the horizontal threshold line of 1.3 (-log of p value=0.05). Genes with large fold-change values would lie to the left (downregulated genes) or right (upregulated genes) of a vertical threshold line. Therefore, significantly upregulated or downregulated genes identified by *t-*tests would be located in the upper left or upper right parts of the plot.

Of the 204 differentially expressed genes, those non-hypothetical genes with known functions were classified into functional groups according to the Entrez Gene database and literature review (Appendix 1). A total of 131 differentially expressed genes were classified into 22 functional categories: cell receptors (5%), cell signaling and extracellular communication proteins (7%), intracellular transducers, effectors, and modulators (12%), metabolism (12%), and transcription (14%). Among the relatively downregulated genes were cell receptors, including *PTPRB* (p=0.0384), and relatively upregulated genes included transcripts for growth factors, including *FGF-2* (p=0.0161), and *TGFBI*/*βig-h3* (p=0.0395).

### Global pathway analysis

**Figure 5:**
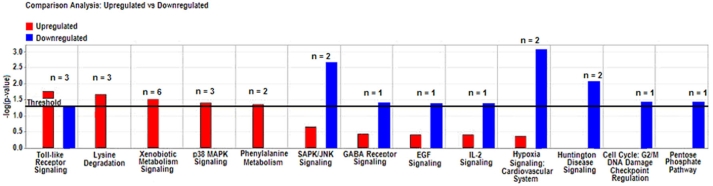
Global canonical pathway analysis of significantly upregulated and downregulated genes in minus lens-treated eyes as compared with plus lens-treated eyes. Data sets were analyzed by the Ingenuity Pathways Analysis software (Ingenuity^®^ Systems). The significance is expressed as a p value that is calculated using the right tailed Fisher exact test. Red and blue bars represent significantly upregulated and downregulated genes associated with canonical pathways, respectively. In the figure, n represents the number of genes identified in each of the pathways.

Global canonical pathway analyses were employed to identify the cellular mechanisms underlying the ocular response to minus lens-induced and plus lens-induced defocus. The 204 genes that were significantly altered in their expression levels (upregulated and downregulated) during lens treatment were submitted for global canonical pathway analysis (Ingenuity Pathways Analysis, Ingenuity Systems). Several genes that showed significant changes in gene expression in the choroid/RPE of minus lens-treated eyes as compared with plus lens-treated eyes were identified in Ingenuity’s library of canonical pathways (Figure 5). The significance calculated for each canonical pathway is a measurement of the likelihood that the pathway is associated with the upregulated or downregulated genes by random chance. Five canonical pathways (toll-like receptor signaling, lysine degradation, xenobiotic metabolism signaling, p38 mitogen-activated protein kinase (MAPK) signaling, and phenylalanine metabolism) showed significant upregulation. In contrast, stress-activated protein kinase (SAPK) and c-Jun NH_2_-terminal kinase (JNK) signaling, GABA receptor signaling, epidermal growth factor (EGF) signaling, interleukin-2 (IL-2) signaling, hypoxia signaling, Huntington disease signaling, cell cycle: G2/M DNA damage checkpoint regulation, and pentose phosphate pathways showed significant downregulation. Hypoxia signaling in the cardiovascular system and SAPK/JNK signaling pathways were found to be the most significantly perturbed canonical pathways (p<0.01) in minus lens-treated eyes as compared with plus lens-treated eyes. These results suggest that molecular events characterized for these canonical pathways may also be involved in mediating the choroid/RPE response to visually induced changes in ocular growth.

### Validation of genes of interest

**Figure 6:**
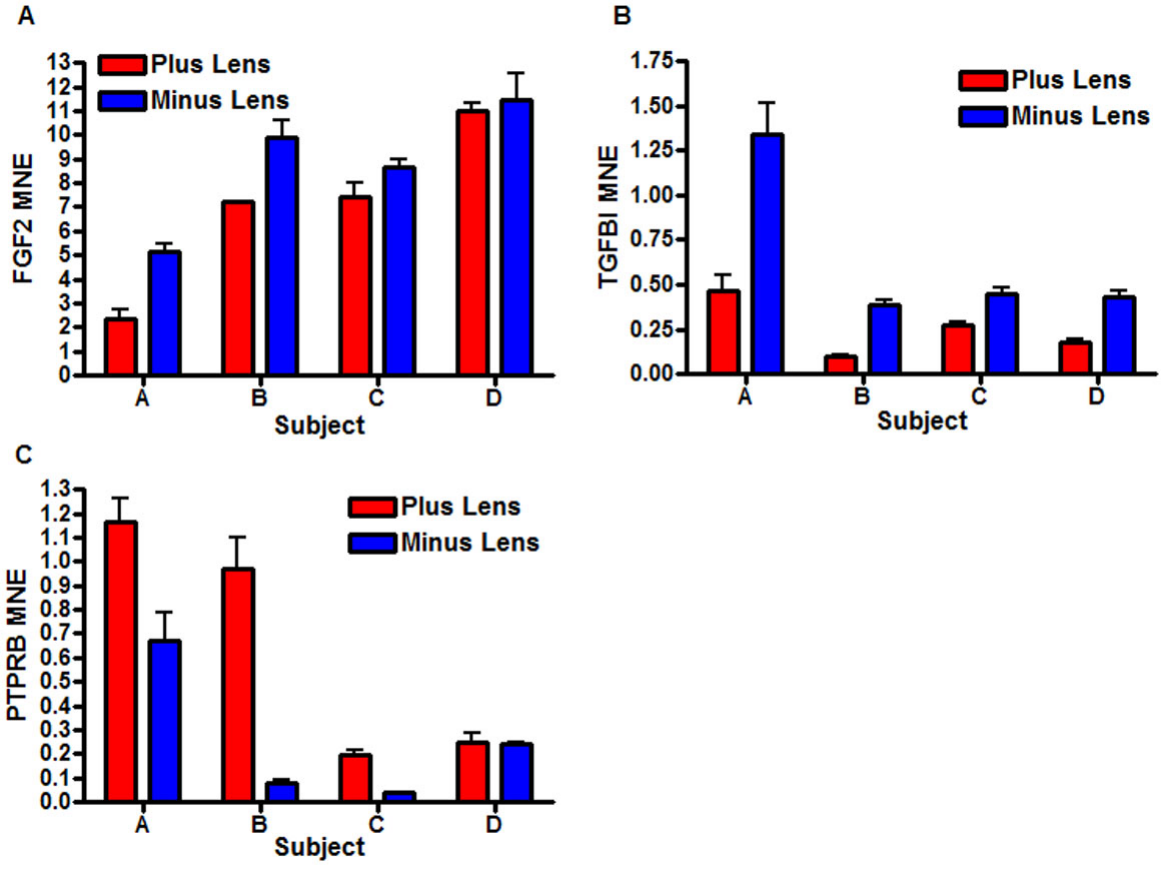
Real time polymerase chain reaction quantification of differentially expressed genes in the marmoset choroid/RPE. The expression of fibroblast growth factor-2 (**A**), transforming growth factor beta induced (**B**), and protein tyrosine phosphatase, receptor type, B (**C**) mRNA was measured using human gene-specific primers (Appendix 1). Graphs represent the mean normalized expression of each gene of interest in individual marmoset choroid/RPE samples following minus or plus lens-treatment. All reactions were run in triplicate and normalized to the reference gene, cyclophilin A.

**Figure 7:**
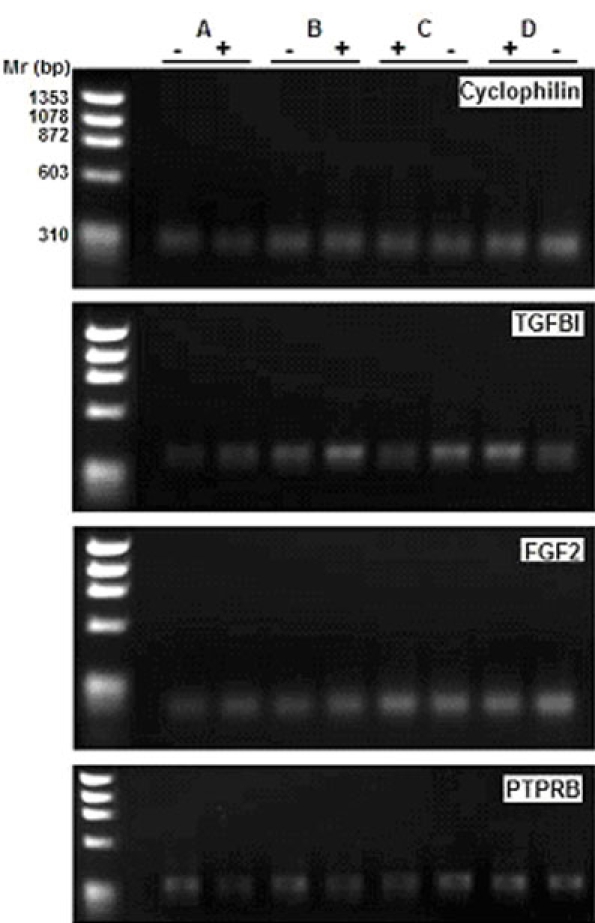
Real time polymerase chain reaction (qRT-PCR) of choroid/RPE genes. Genes identified on microarrays as significantly differentially expressed in choroid/RPE of minus lens-treated eyes as compared with plus lens-treated eyes were assessed by qRT-PCR. cDNA was prepared from total RNA extracted from choroid/RPE of +5 D lens-treated (+) and −5 D lens-treated (-) eyes of subjects **A, B, C**, and **D**. Human primers were used to amplify 172-373 bp regions of cyclophilin A (*cyclophilin*), transforming growth factor, beta-induced (*TGFBI*), 68 kDa, fibroblast growth factor -2 (*FGF2*), and protein tyrosine phosphatase, receptor type, B (*PTPRB*; see Table 1).

To validate choroid/RPE gene expression differences in minus lens-treated versus plus lens-treated eyes undergoing changes in ocular growth rates, we performed quantitative real-time PCR on three genes of interest. Due to limitations in RNA from marmoset lens-treated eyes available for real-time PCR, genes were selected based on their potential for regulating ocular growth and cell-matrix interactions during periods of accelerated or decelerated ocular growth (e.g., a secreted growth factor or extracellular matrix molecule). Therefore, the significantly upregulated genes, *TGFBI* and *FGF-2*, were selected for confirmation. Additionally the tyrosine phosphatase receptor, *PTPRB*, was selected for real-time PCR experiments as this was one of relatively few significantly downregulated genes in the choroid/RPE of minus lens-treated eyes as compared with plus lens-treated eyes. Using the same eight samples of total choroid/RPE RNA used for microarray analyses, quantitative real-time PCR analyses confirmed that *PTPRB* was significantly downregulated in the choroid/RPE of minus lens-treated eyes as compared with plus lens-treated eyes (−54.6%, p<0.05, paired *t*-test) while *TGFBI* and *FGF-2* were significantly upregulated in the choroid/RPE of minus lens-treated eyes as compared with plus lens-treated eyes of the four marmosets examined (+139.7%, +43.7%, respectively p<0.05, paired *t*-test; Figure 6). DNA gels confirmed the correct primer size (Figure 7). Melt curve analyses confirmed a lack of primer dimer formation and sequencing of the PCR product confirmed amplification of the gene of interest (Table 1).

It should be noted that the magnitude of the differences in gene expression observed on oligonucleotide microarrays (Appendix 1) was substantially different as compared with real-time PCR results. Numerous methodologic differences can influence the magnitude of the changes in gene expression observed on oligonucleotide microarrays and real-time PCR, including different efficiencies of reverse transcriptases and priming methods for real time PCR and microarray experiments and differences in data normalization between microarray analysis and real time PCR [32]. It was the approach of the present study to identify differentially regulated genes in choroid/RPE from eyes following treatment with plus and minus lenses regardless of the differences in magnitudes of the relative gene expression. Using this approach, candidate genes may be identified that are involved in the regulation of ocular growth.

Interestingly, relative gene expression changes for *FGF-2* and *PTPRB* in subject D were lower than the other three subjects and subject D exhibited the most negative refractive error in the plus and minus lens-treated eyes at the end of the 92-day lens treatment period (Figure 1). While these data suggest that subject D responded differently to the binocular aniso lens treatment, we cannot exclude the possibility that these responses are within the normal range of variation. Changes in vitreous chamber depth (Figure 1B) and *TGFBI* expression (Figure 6B) for subject D were similar to that of the other three subjects.

### Transforming growth factor β-induced protein in marmoset choroid/RPE

**Figure 8:**
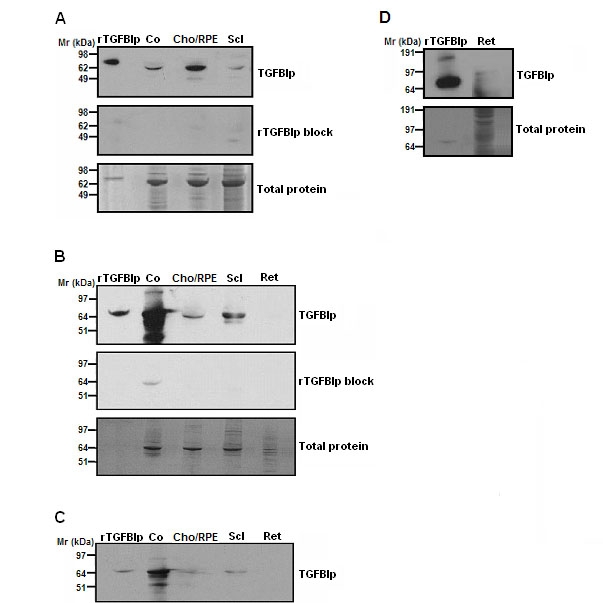
Transforming growth factor, beta-induced protein detection in marmoset and human ocular tissues. Western blot analysis identified the presence of transforming growth factor, beta-induced protein (TGFBIp) in the cornea (Co), choroid/RPE (Cho), sclera (Scl), and retina (Ret) of the marmoset (**A**) and human eye (**B**). Pre-incubation of TGFBIp antibodies with recombinant TGFBIp protein (rTGFBIp; 1 µM final concentration) abolished all major anti-TGFBIp immunoreactive bands in marmoset tissue extracts, with the exception of a faint band in the human cornea lane migrating at ≤ 65 kDa (**B**, center panel; rTGFBIp block). Total protein loaded in each well was visualized by Coomassie Blue staining (**A** and **B**, bottom panels). **C:** Lighter exposure of Figure 7B (top panel). Recombinant TGFBIp protein (rTGFBIp, 40 ng) and cornea served as positive controls.

Based on microarray and quantitative real-time PCR results, TGFBI was selected for further study. This gene was significantly upregulated in the choroid/RPE of minus lens-treated eyes relative to plus lens-treated eyes and the expressed protein, TGFBIp, has not been previously identified in ocular tissues other than the cornea [33,34]. Western blot analysis was used to examine the distribution of TGFBIp in the cornea, choroid/RPE, and sclera of the marmoset (Figure 8A) and in the cornea, choroid/RPE, retina and sclera of the human eye (Figure 8B). A single band of about 65 kDa could be identified in marmoset protein extracts of cornea, choroid, and sclera (Figure 8A, upper panel), which was abolished following pre-incubation of primary antibody with human recombinant TGFBIp (rTGFBIp block; Figure 8A, center panel), suggesting that these bands represent the TGFBIp protein in these tissues. TGFBIp was not detected in the human or marmoset retina (Figures 8B and 8D). Western blot analysis of human corneal extracts also demonstrated a major TGFBIp-immunopositive band of about 65 kDa as well as a minor band of about 51 kDa (Figure 8B, upper panel and Figure 8C), while a 65-70 kDa doublet was apparent in scleral extracts. A single band of about 65 kDa was apparent in human choroidal extracts. The single 65 kDa bands as well as the 65-70 kDa doublets observed in human cornea, choroid and scleral extracts were abolished following pre-incubation of primary antibody with human recombinant TGFBIp (rTGFBIp block; Figure 8B, middle panel), suggesting that these bands represent the TGFBIp protein in these tissues. The doublet observed in the scleral sample most likely represented the native and processed forms of TGFBIp, as described for human neuroblastoma cells [35].

### The effect of transforming growth factor β-induced protein on scleral fibroblast cell attachment

**Figure 9:**
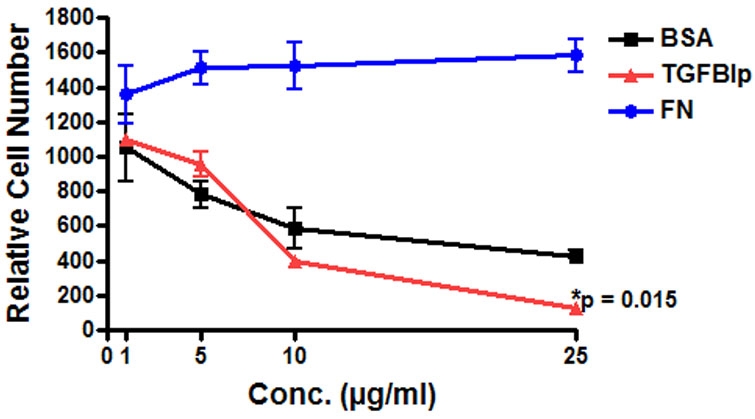
Inhibition of cell attachment by transforming growth factor, beta-induced protein. Solid-phase adhesion assay of primary human scleral fibroblasts to wells pre-coated with 0-25 μg bovine serum albumin (BSA), fibronectin (FN), and transforming growth factor, beta-induced protein (TGFBIp) on poly-D-lysine 96-well plates. Cells were seeded 2000 cells/well and allowed to attach for 45 min in serum-limiting media. Unattached cells were gently rinsed off and remaining attached cells were quantified by measuring hexosaminidase, as described in Methods. Cell attachment was significantly decreased in wells coated with 25 μg TGFBIp as compared to BSA controls. Data are expressed as the mean±SEM by the Student *t*-test for unmatched pairs for three individual experiments in triplicate. In the figure, the asterisk indicates p≤0.05.

Cell attachment assays were used to gain insights on the functional significance of increased *TGFBI* expression in the choroid/RPE of minus lens-treated eyes relative to plus lens-treated eyes. The attachment of HSFs to poly-D-lysine-coated plates was examined after treatment of wells with varying concentrations of TGFBIp, fibronectin, and BSA (Figure 9). Pretreatment of wells with TGFBIp at a concentration of 25 μg/ml (0.38 μM) significantly decreased the binding of human scleral fibroblasts, as compared with equivalent concentrations of BSA (-70.7%; p<0.05) and fibronectin (-92.1%; p<0.001). Cell attachment was slightly, but not significantly, inhibited by TGFBIp at a concentration of 10 μg/ml as compared with BSA (-32.6%, p*=*0.246). In contrast, scleral fibroblast attachment to fibronectin substantially increased as compared with equivalent concentrations of BSA (+93%, p<0.01 for 5 μg/ml; +158.9%, p<0.001 for 10 μg/ml, and 270.6%, p<0.01 for 25 μg/ml, respectively).

## Discussion

Changes in choroidal thickness, permeability, and protein synthesis are associated with changes in visually guided ocular growth rates in chicks [14,15] and primates [16,17]. Moreover, co-culture of chick choroids or choroidal fluid with chick sclera has indicated that the choroids release bioactive factors that inhibit scleral proteoglycan synthesis [21,36]. Based on these observations, we hypothesized that changes in choroid/RPE gene expression were responsible for the production of secreted proteins that could regulate the rate of scleral proteoglycan synthesis and ocular growth.

Microarray platforms (Atlas™ Plastic Human 12K Array, Clonetech Labs) were employed to identify gene expression profiles in choroid/RPE complexes isolated from primate eyes treated with +5 D lenses as well as fellow eyes treated with −5 D lenses. By comparing gene expression profiles from eyes treated with +5 D lenses with gene expression profiles of eyes treated with −5 D lenses, we anticipated that choroidal genes would be identified in marmosets that would help to elucidate choroidal and RPE responses to imposed defocus and could lead to the identification of choroidal and or RPE proteins that play a role in scleral extracellular matrix organization and the regulation of ocular growth.

Binocular +5/-5 D lens treatment was used in our investigation to maximize differences in interocular growth rates and the refractive states of the paired eyes to identify changes in gene expression in choroid/RPE associated with different ocular growth states. The application of this binocular lens paradigm has been well described and shown to be both an effective and reproducible method to induce anisometropia [37,38], which is greater than that observed with monocular plus or minus lens treatment (unpublished). In the present study, measurements of ocular growth and refraction were made every two weeks which enabled us to determine the rate of ocular growth in plus and minus lenses throughout the treatment period. Following 92 days of lens wear binocular +5/-5 D lens treatment resulted in distinct anisometropia, with significantly higher negative refractive errors and significantly longer vitreous chamber depths in minus lens-treated eyes as compared with plus lens-treated eyes. Analyses of vitreous chamber depths and refractive changes during this treatment period indicated that ocular growth and refractive states had not stabilized in either the positive lens-treated or negative lens-treated eyes following 92 days of lens wear. Lens-treated eyes continued to exhibit changes in growth and refraction at the end of the 92 day treatment period. Therefore, we cannot exclude the possibility that choroid/RPE gene expression changes identified in this study are directly involved in the ocular signaling cascade resulting from aniso-lens treatment, or represent an epiphenomenon, i.e., as a consequence of lens-induced changes in ocular growth.

While several recent reports have described changes in retina and retina/RPE gene expression associated with the development of myopia [25,39], no previous studies have examined gene expression changes in the choroid and RPE during visually induced changes in ocular growth. In the present study, microarray analysis identified 204 (~1.7%) genes that were significantly differentially expressed on an 11,867 gene human array. The majority of differentially expressed genes (~90%) were upregulated in choroid/RPE's of minus lens-treated eyes as compared to plus lens-treated eyes (or downregulated in plus lens-treated eyes as compared with minus lens-treated eyes). These microarray results suggest that gene expression profiles of choroid/RPE from plus lens-treated eyes are distinct from that of minus lens-treated eyes, and differentially expressed genes are likely to be relevant to the mechanism of ocular compensation for induced refractive error.

Interestingly, hypoxia signaling in the cardiovascular system and SAPK and JNK signaling pathways were found to be the most significantly perturbed canonical pathways (p<0.01), due to significant downregulation of JUN (GenBank J04111) and upregulation of tumor protein p53 (TP53; GenBankM14694) genes in choroid/RPE's of minus lens-treated eyes relative to expression levels in plus lens-treated eyes. Interestingly, hypoxia-induced activation of tumor-related proteins such as extracellular signal-regulated kinase, JNK, and p53 has been shown to be nitric oxide-mediated in a variety of experimental systems [40,41]. Previous studies in chicks have suggested that nitric oxide may be involved in the choroidal response to myopic defocus, since treatment with the nitric oxide synthase inhibitor, L-NAME, transiently inhibited the expected increase in choroidal thickness [42]. Taken together, these results suggest that the molecular signaling pathways in the choroid/RPE associated with +5 D lens-treatment may be similar to those involved in hypoxia-induced signaling pathways.

In the present study, we examined the expression of genes that have been previously implicated to play a role in myopia development or have been previously characterized to be involved in cell-matrix interactions and extracellular matrix remodeling. Due to limited mRNA following microarray analysis only three genes (*FGF-2*, *TGFBI*, and *PTPRB*) were reevaluated in the four individual marmosets by quantitative real-time PCR. Although the magnitude of the expression differences between plus and minus lens-treated eyes varied between individual marmosets as well as that obtained from microarray and real-time PCR results, significant changes in the expression of these three genes were detected in the choroid/RPE of the four marmosets examined in this study.

FGF-2 is a pro-mitogenic growth factor located in basement membranes and in the subendothelial extracellular matrix of blood vessels and is involved in wound healing, tumor formation, and angiogenesis [43,44]. *FGF-2* mRNA and protein expression have been previously described in the retina [45,46], choroidal endothelial cells [47], and sclera [46,48]. Studies in chicks have suggested that FGF-2 acts together with TGF-β1 to regulate ocular growth [49,50], possibly through the alteration of retinal circuitry that controls postnatal ocular growth [51]. Although scleral and retinal levels of FGF-2 have been reported to be unchanged during the development of myopia or recovery from myopia in the tree shrew, the expression of the FGF-2 receptor, FGFR-1, has been shown to be upregulated in the sclera of myopic tree shrew eyes and downregulated to control eye levels during recovery, suggesting that FGF-2 plays a role in the control of scleral remodeling during visually guided ocular growth [48]. Taken together, these results and the results of the present study suggest that FGF expression is altered in the choroid/RPE during lens-induced changes in ocular growth and may play a role in chemical cascades in the retina, choroid, and sclera.

In addition to *FGF-2*, the expression of the TGFβ1-inducible gene *TGFBI* was elevated in eyes actively elongating after minus lens-treatment, relative to that of plus lens-treated eyes. TGFBIp is a secreted extracellular matrix protein first isolated from human lung adenocarcinoma cells following treatment with TGF-β1 [52]. Since its discovery, TGFBIp, has been demonstrated to have a physiologic as well as a pathologic role in other cell types including human melanoma cells, human mammary epithelial cells, human keratinocytes, human foreskin fibroblasts, and porcine articular chondrocytes following stimulation with TGF-β1 [53-56] as well as in cultures of corneal epithelial cells [57,58], primary human foreskin fibroblasts [54], human bladder smooth muscle cells [59,60], fibroblast-like synoviocytes [61], and primary HSFs [62]. Depending on the cell type under study, TGFBIp has been shown to promote [60,63-68] or inhibit [53,69] cell adhesion through integrin-mediated mechanisms. Results presented here confirmed the presence of TGFBIp protein in the cornea, choroid/RPE, and sclera of the marmoset and human eye and suggest that TGFBIp inhibits scleral fibroblast cell adhesion to poly-D-lysine-coated plates. Similar results have been observed when HSFs were plated on collagen type I-coated plates in the presence of TGFBIp (unpublished). Taken together, we speculate that increased synthesis of TGFBIp by the choroid/RPE may participate in modulating the biomechanical properties of the sclera through alterations in cell-matrix interactions.

The finding that *TGFBI* expression was increased in minus lens-treated eyes relative to that of plus lens-treated eyes suggests a role for TGF-β in mediating the ocular responses during visually guided ocular growth. However, studies evaluating TGF-β in a variety of ocular tissues during the development of myopia have been inconclusive. One study reported increased TGF-β2 in both the retina, RPE, choroid, and sclera of myopic chicks eyes [50] while others reported decreased in TGF-β levels in the retina, retinal pigment epithelium, and choroid during the development of myopia in chicks [70] as well as decreased RNA levels of the *TGF-β* isoforms, *TGF-β1*, *TGF-β2*, and *TGF-β3* in the tree shrew sclera during myopia development [11].The finding of increased *TGFBI* mRNA in the choroid of marmoset eyes during the development of myopia may represent a downstream response to a TGF-β mediated cascade occurring in the retina, choroid or sclera. Alternatively, the relative upregulation of *TGFBI* in actively elongating eyes may represent a downstream response to another cytokine since TGFBIp has also been shown to be induced by interleukin-1β and tumor necrosis factor α in fibroblast-like synoviocytes isolated from rheumatoid arthritis synovial tissue [61].

Additionally, we elected to use real-time PCR to confirm the changes in expression of transmembrane receptor-like protein, *PTPRB*, on the basis that it was one of the few genes to be significantly downregulated in minus lens-treated eyes as compared with plus lens-treated eyes. Real time PCR confirmed that *PTPRB* was significantly downregulated (−54.6%) in minus lens-treated eyes as compared with plus lens-treated eyes of the four marmosets examined. Members of the PTP family are highly specific, tightly regulated enzymes with important regulatory roles that have been implicated in a variety of cellular processes including cell growth, differentiation, mitotic cycle, and oncogenic transformation [71-73]. Interestingly, *PTPRB* expression has also been shown to regulate umbilical vein endothelial cell migration and tube formation [74]. Based on these results, we speculate that the relative downregulation of *PTPRB* in the choroids of minus lens-treated eyes may represent a response by choroidal vascular endothelial cells to regulate choroidal vascularization during the development of myopia.

Several recent microarray studies have demonstrated significant gene expression changes in the retina or retina and RPE following form deprivation in the macaque [75], green monkey [75], mouse [76], and chick [39] eyes. The expression of vasoactive intestinal peptide, cyclin A2, and hepatoma-derived growth factor were shown to be upregulated in retinas of form-deprived primate eyes and associated with increased (retinal) cell proliferation [75]. Analysis of retinal gene expression changes in form deprived mouse eyes demonstrated reduced expression of the immediate-early genes *Egr-1* and *cFos* mRNA and temporarily increased expression of the putative oncogene, Akt2 and the JNK binding protein, Mapk8ip3 [76]. In contrast, analyses of chick retina/RPE during the development of form deprivation myopia demonstrated only minimal changes in retinal gene expression, including downregulation of bone morphogenetic protein 2, vasoactive intestinal peptide, preopro-urotensin II-related peptide and mitogen-activated protein kinase phosphatase 2, and upregulation of endothelin receptor type B and interleukin-18 [39].

Changes in RPE gene expression in response to +10 D and −10 D lenses in young chicks have been recently reported [26]. Among 157 upregulated genes and 632 downregulated in minus lens-treated eyes as compared to contralateral control eyes, the expression of *TGF-β3, SMAD2*, and *SMAD3* was downregulated in RPE from minus lens-treated eyes as compared with fellow eyes, whereas TGF-β receptor type I (*TGFBR1*), TGF-β receptor type III (*TGFBR3*), somatostatin (*SST*), insulin receptor substrate 1 (*IRS1*), and insulin-like growth factor-binding protein 1 (*IGFBP1*) were shown to be upregulated in RPE from minus-lens treated eyes. Interestingly, we did not detect differences in the expression of these genes in the choroid/RPE of binocular +/− lens-treated marmoset eyes. It is possible that the inclusion of the choroid in the microarray analysis of the present study may have masked these RPE gene expression changes, if present. However, considering the differences in retinal gene expression observed during myopia development in chicks, mice, and primates, it is also possible that the retina, RPE, choroid, and sclera signaling cascades are distinct in chicks as compared with primates, due to fundamental structural differences in the sclera and the nature of scleral response during visually guided ocular growth [4].

Treatment of young marmosets with binocular aniso-lenses (+5/-5 D) was highly effective in generating large interocular differences in vitreous chamber depth and refraction. However, we acknowledge that this aniso-lens paradigm limits gene expression analyses to the identification of relative changes in gene expression between the plus lens and minus lens-treated eyes, rather than the identification of specific gene expression changes associated with plus lens-induced and minus lens-induced defocus. We also acknowledge the technical limitations of our microarray analysis, which include the use of human primers for marmoset tissues, and the use of complex tissues to detect gene expression changes. As mentioned, some gene expression changes in some cell types in the choroid/RPE may go undetected due to cancelling out by opposite changes in gene expression by different cell types. Nevertheless, gene expression profiling and pathway analyses of the microarray data obtained from this study have identified potential candidate myopia genes for more focused future studies in animal models as well as in humans and have provided a detailed knowledge of changes in choroidal and RPE gene expression during periods of accelerated and decelerated ocular growth. This detailed knowledge will provide a basis for understanding the molecular mechanisms underlying the ocular response to imposed defocus and will help to elucidate the role of the choroid/RPE in the regulation of visually guided ocular growth.

## Appendix 1: Differentially expressed genes in the choroid/RPE's of binocular lens-treated marmoset eyes

To access the data, click or select the words “Appendix 1”. This will initiate the download of a pdf file.

Fold change was calculated as the ratio of the average magnitude of gene expression in the minus lens-treated eyes over that of the plus lens-treated eyes (positive numbers; relative upregulation) or the magnitude of gene expression in the plus lens-treated eyes over that of the minus lens-treated eyes (negative number; relative downregulation).
